# Pilot Testing Two Versions of a Social Network Intervention to Increase HIV Testing and Case-finding among Men in South Africa’s Generalized HIV Epidemic

**DOI:** 10.3390/ijerph21010054

**Published:** 2023-12-30

**Authors:** Leslie D. Williams, Alastair van Heerden, Xolani Ntinga, Georgios K. Nikolopoulos, Dimitrios Paraskevis, Samuel R. Friedman

**Affiliations:** 1 Division of Community Health Sciences, University of Illinois Chicago School of Public Health, Chicago, IL 60612, USA; 2 Sweetwaters Centre for Community Based Research, Human Sciences Research Council, Pietermaritzburg 3201, South Africa; 3 Medical School, University of Cyprus, Nicosia 1678, Cyprus; 4 Department of Hygiene, Epidemiology and Medical Statistics, Medical School, National and Kapodistrian University of Athens, 11528 Athens, Greece; 5 Department of Population Health, New York University School of Medicine, New York, NY 10016, USA

**Keywords:** HIV case-finding intervention, social networks, HIV testing, South Africa

## Abstract

Locating undiagnosed HIV infections is important for limiting transmission. However, there is limited evidence about how best to do so. In South Africa, men have been particularly challenging to reach for HIV testing due, in part, to stigma. We pilot-tested two versions of a network-based case-finding and care-linkage intervention. The first, TRIP, asked “seeds” (original participants) to recruit their sexual and/or injection partners. The second, TRIPLE, aimed to circumvent some stigma-related issues by asking seeds to recruit anyone they know who might be at risk of being HIV-positive-unaware. We recruited 11 (18% male) newly diagnosed HIV-positive (NDP) seeds from two clinics in KwaZulu-Natal, South Africa and randomly assigned them to either TRIP or TRIPLE. Network members were recruited two steps from each seed. The TRIP arm recruited 12 network members; the TRIPLE arm recruited 62. Both arms recruited NDPs at higher rates than local clinic testing, with TRIP (50.0%) outperforming (*p* = 0.012) TRIPLE (14.5%). However, TRIPLE (53.2%) was far superior to clinics (27.8%) and to TRIP (25.0%) at recruiting men. Given challenges around testing and treating men for HIV in this context, these findings suggest that the TRIPLE expanded network-tracing approach should be tested formally among larger samples in multiple settings.

## 1. Introduction

Locating undiagnosed HIV infections (via HIV testing) is the important first step of the 95–95–95 strategy of limiting HIV transmission [1]. Locating men for HIV testing is a particularly important challenge in many parts of the world, since poorer engagement of men with HIV services, including testing, is currently a widely discussed concern in the HIV prevention field [2], pertaining to many parts of the world, and to Sub-Saharan Africa in particular. In South Africa, national models estimate that 8–15% of HIV infections are undiagnosed [3,4,5], but with a large gender-based disparity: up to 21.8% of HIV infections among men are estimated to be undiagnosed, while only up to 11.3% of HIV infections among women are estimated to be undiagnosed [4]. Models have also estimated that, among HIV-positive men in South Africa, 30% to 80% (depending on age at the time of seroconversion) will experience disease progression (CD4+ count less than 350 cells) before diagnosis [6,7]. The gender differences in the treatment cascade leading to these disparate outcomes begin with disparities in HIV. Among Department of Health clinics in the district closest to the study site, on average, approximately 28–36% of HIV testing patients are male [8]. This underrepresentation of men is important to address not only because of the importance of improving the health of men who are already HIV-positive but also as a means of reducing new HIV infections. One study estimated that, nationally in South Africa, male-to-female transmission of HIV comprises two thirds of all HIV transmission [9].

Factors thought to partially explain this gender disparity in HIV testing and other types of HIV-related health services include gender norms and practices (i.e., constructions of hegemonic masculinity [2,10,11,12]) that may influence men’s willingness to be vulnerable in front of women (including healthcare providers); their views of healthcare settings as female spaces; their fears of being seen as weak or unhealthy [2]; and their degree of willingness to speak openly with women, including both their healthcare providers and their own sexual partners about HIV testing and HIV status.

Issues of HIV-related stigma may also disproportionately affect men in South Africa (and in other settings). Men may be viewed as more likely to have multiple concurrent partnerships; and may be viewed as the perpetrators of HIV transmission and sexual violence [2,13,14]. Fear of or desire to avoid such gender-based stigma may engender or exacerbate reluctance among men not only to seek HIV testing in traditional clinic settings, but also to discuss HIV testing or serostatus with their sexual partners.

While these challenges and barriers are well-documented, there is limited evidence about how best to facilitate men’s care by extending the reach of current case-finding methods to include more men, particularly within generalized epidemics. Network-tracing case-finding interventions that focus on tracing the networks of people with recent HIV infection (i.e., people infected with HIV in the last six months) have been found to be successful among concentrated epidemics. Specifically, the Transmission Reduction Intervention Project (TRIP), which traced the risk partners of recently HIV-infected people who inject drugs in Greece and Ukraine, and of recently infected men who have sex with men in the United States, was found to efficiently locate both additional recently infected people [15,16] and people with longer-term undiagnosed HIV infections [17,18].

There have been a number of intervention studies focused on primary sex partner testing in South Africa [19,20,21,22], but to our knowledge, full risk network recruitment to in-person HIV testing has not yet been tested there. Furthermore, in South Africa, gender norms and constructions of masculinity may limit men’s willingness to disclose their HIV status to their sexual partners, or to discuss HIV testing at all with their sexual partners. This may limit the ability of primary partner interventions and traditional risk network-tracing strategies that trace the direct sexual and/or injection contacts of HIV-positive individuals to successfully recruit men to HIV testing. A modification or adaptation of network-tracing strategies developed specifically for the South African context may be needed to overcome or circumvent some of the specific challenges related to stigma and/or gender norms that may work against sexual partner tracing as a basis for recruitment to HIV testing.

This study therefore presents pilot test data from a test conducted in South Africa of two different versions of the TRIP network-tracing HIV case-finding intervention. The first version tested was the original TRIP approach, which asks “seeds” (original participants) to recruit all of their sexual and/or injection partners to the study for HIV testing (and linkage to treatment). The second version tested was a version of TRIP adapted specifically for the South African setting based on focus group data. This adapted version, called TRIPLE (Transmission Reduction Intervention Project—Linkage-Expanded model), aims to circumvent some issues of stigma and some gender norms and practices that could potentially limit the ability of this traditional risk network-tracing method to recruit men. Specifically, it provides seeds with education on HIV transmission and risks, and then asks them to recruit members of their *extended* social networks—i.e., anyone they know, including their sexual and/or injection partners—whom they think might be HIV-positive but undiagnosed.

The present study aims to address the following questions using pilot study data collected for a small network-based sample in KwaZulu-Natal:

(1) Which version of the intervention recruited more men for HIV testing?
(2) Which version of the intervention located higher rates of previously undiagnosed HIV-positive network members (i.e., newly diagnosed HIV-positive participants, or NDPs)? Did this vary by gender?

Addressing these questions will serve to provide preliminary evidence towards identifying the most appropriate case-finding intervention model to test on a larger scale in this context.

## 2. Materials and Methods

### 2.1. Setting

The study setting is a sub-district of KwaZulu-Natal (KZN) province in South Africa that includes a mid-size city. It is situated within the UMgungundlovu Department of Health District, comprised of 55 clinics and located in the Msunduzi region. This region contains urban, suburban, and rural locations. It is comprised mostly of isiZulu- and English-speaking populations of Zulu heritage but includes some areas with large white and Indian populations. Located about 150 km from Durban, it is characterized by low per capita income (40% of households reported an annual income < $1050 during the most recent Census [23]) and high unemployment (43% in 2020 [24]). HIV prevalence in KZN overall was estimated at 27% in 2017 [25].

### 2.2. Sample

We recruited newly diagnosed HIV-positive “seeds” (i.e., original participants who were referred to us by clinics from “business as usual” HIV testing). We asked clinic staff at two Department of Health clinics in KwaZulu-Natal to refer to us any person at least 18 years of age whom the clinic newly diagnosed with HIV during our approximately 11-week seed recruitment period starting in January 2018. Newly diagnosed HIV-positive (NDP) seeds were defined as adults who tested positive for HIV at a study clinic, had no recorded previous positive HIV test, and indicated to clinic staff that they had never received a positive HIV test result before. Clinics were selected based on their relative geographic centrality to and representativeness of the broader Department of Health district that we hope to include in a larger future efficacy trial. Clinic-referred (18+ year-old) NDP were eligible to be seeds if they were able to understand and be interviewed in isiZulu or English and were able to give written informed consent.

We also recruited network members for all enrolled seeds. (Network member type varied as a function of study arm; see Section 2.3 below.) Seeds were given recruitment coupons to recruit their network members by distributing coupons to them. Coupons contained unique, confidential alphanumeric codes with no identifying information. These codes were used to link seeds’ network members to them for analytic purposes. Network members referred to us by seeds were eligible if they presented a recruitment coupon that we had distributed to a study participant less than two months ago, were at least 18 years old, able to understand and be interviewed in isiZulu or English, and able to give written informed consent. Interviews were conducted either in isiZulu or in English, according to participant preference.

A “two steps” algorithm of network member recruitment was followed, meaning that all participants who were recruited by seeds (Step 1) were also asked to help us recruit additional participants (Step 2) who were their own additional network members.

### 2.3. Intervention

Seeds were randomly assigned to either the TRIP intervention arm or to the TRIPLE intervention arm by using randomization software to randomly assign alphanumeric codes on each study recruitment coupon to either the TRIP or TRIPLE study arm, before giving the coupons to clinic staff to distribute to prospective seed participants. Clinic staff were blind to the assignment purposes of the alphanumeric codes. All members of each seed’s network were automatically considered to be a part of the study arm to which the seed was randomly assigned. In the TRIP intervention arm, we asked seeds to recruit all of their direct sexual and/or injection partners (i.e., anyone they had sex with or injected with in the last six months) to participate in the study and to get tested for HIV. The number of recruitment coupons given to each seed corresponded to the number of sexual and/or injection partners he or she reported. The participants they recruited (Step 1 network members) received HIV testing and counseling (HTC) compliant with the South African national Department of Health’s HTC guidelines, were interviewed, and were asked to recruit all of their additional sexual and/or injection partners from the last six months (Step 2 network members). Step 2 network members who participated received HIV testing and counseling and were interviewed but were not asked to recruit their own network members. All participants asked to recruit network members were told that they did not have to disclose their HIV status to the people they recruited if they did not want to, and that they should use whatever language they were comfortable with when recruiting others to the study. All participants who tested HIV-positive were referred to care at Department of Health clinics and were offered assistance with making appointments there.

In the TRIPLE intervention arm, we used a diagram to educate participants about HIV transmission risks and how HIV infection is transmitted through networks, and then asked them to recruit anyone they knew (both sexual/injection partners and others such as friends and family members) whom they thought might be at risk of being HIV-positive but unaware based on the education we provided them and their knowledge of their network members’ behaviors and testing history. We asked them to tell us how many people they might want to recruit given these instructions. We then gave them recruitment coupons for the network members they counted, plus 5 extra coupons for people they forgot to count or thought of later. Recruited network members (Step 1) received HIV testing and counseling (HTC) compliant with the South African national Department of Health’s HTC guidelines and were interviewed and asked to recruit their own network members, following the same brief education and instructions that the seeds received. Step 2 network members received HIV testing and counseling and were interviewed, but were not asked to recruit their own network members. All participants asked to recruit network members were told that they did not have to disclose their HIV status to the people they recruited if they did not want to, and that they should use whatever language they were comfortable with when recruiting others to the study. All participants who tested HIV-positive were referred to care at Department of Health clinics and were offered assistance with making appointments there.

All study materials and procedures were approved by a research ethics committee at Human Sciences Research Council in South Africa, and by an institutional review board at National Development and Research Institutes in New York, United States.

### 2.4. Measures

NDPs were defined as participants who test HIV-positive, have no recorded previous positive HIV test (in the case of seeds who were tested at Department of Health clinics), and indicate to clinic staff (in the case of seeds) or to study staff (in the case of network members) that they have never received a positive HIV test result before.

Gender was measured using participant self-report. All participants reported being either male (i.e., men) or female (i.e., women).

### 2.5. Data Management and Analysis

Data were all collected electronically on secure mobile devices. Data were encrypted and imported to IBM’s SPSS Statistical Software using a secured network. All data cleaning and data analyses were conducted using SPSS. Rates per seed of the number of male network members recruited for testing were compared between the two study arms (i.e., the TRIP arm was compared to the TRIPLE arm). Rates of the number of NDPs (of either gender) located per seed for the two study arms were also compared. Rates of locating NDP were compared between arms, to local clinics’ average positive test rate of 13%, and by gender for each arm. Chi-square tests were conducted to compare (1) proportions of men recruited in each of the two study arms, and (2) proportions of NDPs located in each of the two study arms.

## 3. Results

### 3.1. Descriptive Statistics of Sample Sociodemographic and Socioeconomic Characteristics

Table 1 presents sociodemographic and socioeconomic characteristics of our full sample of N = 85 participants. Almost 85% of participants reported that isiZulu was their primary language.

### 3.2. Rates of Recruiting Men to HIV Testing

In January 2018, we recruited eleven (two males—18%) newly diagnosed HIV-positive seeds from two clinics in the province of KwaZulu-Natal, South Africa and randomly assigned them to either the TRIP or TRIPLE intervention.

The TRIP arm began with five seeds (three female and two male) and recruited twelve network members total (nine females and three males) across two steps of recruitment of direct sexual (and injection, although no injection was reported) partners. In other words, the TRIP arm recruited men at a rate of 0.6 male network members tested for HIV per seed.

The TRIPLE arm began with six seeds (all female) and recruited 62 network members total (33 males and 29females) across two steps of recruitment of expanded social network members. In other words, the TRIPLE arm recruited men at a rate of 5.5 male network members tested for HIV per seed.

In terms of the proportion of network members tested for HIV who were male, the TRIPLE arm achieved a rate of 53.2%, which was far superior to local Department of Health clinics, which averaged 27.8% men among individuals tested for HIV in early 2018 [8]. Conversely, the TRIP arm performed similarly to business-as-usual testing in local clinics; its network members who were recruited and tested for HIV were comprised of 25% men. Table 2 presents Chi-square analyses comparing the entire TRIPLE sample to the entire TRIP sample in terms of males recruited. Employing a criterion of *p* < 0.10 for significance due to the small sample size of this pilot study, our findings suggest that the TRIPLE arm recruited significantly more men (Χ^2^ = 3.2; *p* = 0.07).

In terms of gender-based recruitment, only one male (including both seeds and network members) in the TRIP arm successfully recruited another participant to the study. On the other hand, almost half (five of eleven) of the males in the TRIPLE arm who were asked to recruit others (seeds and Step 1 network members only as Step 2 network members were not asked to recruit anyone) successfully recruited at least one additional participant to the study. Additionally, 40% of males in the TRIPLE arm were recruited by other males.

### 3.3. Rates of Locating Previously Undiagnosed HIV-Positive Individuals for Linkage to Care

Starting with five NDP seeds, the TRIP arm located six NDP network members, or a rate of 1.2 NDPs located (and referred to treatment) per seed. Starting with six NDP seeds, the TRIPLE arm located nine NDP network members, or a rate of 1.5 NDPs located (and referred to treatment) per seed.

In terms of newly diagnosed cases as a proportion of total network members recruited, both the TRIP and TRIPLE arms recruited higher rates of NDPs than “business as usual” clinic testing among local KwaZulu-Natal clinics given that an average of 13% of tests in the local district clinics in 2017 were positive but not necessarily newly diagnosed positive [8]. However, TRIP’s networks contained higher proportions of NDPs (50.0%) than did TRIPLE’s networks (14.5%). Table 2 presents Chi-square analyses comparing both arms in terms of the proportion of participants recruited who were NDP.

In terms of whether each arm’s success in locating NDPs varied by gender, the TRIP arm located five NDP females and one NDP male (i.e., 20% of NDPs located were male), and NDP males were recruited at a rate of 0.20 per seed. The TRIPLE arm located four NDP males and five NDP females (i.e., 44.4% of NDPs located were male); and NDP males were recruited at a rate of 0.67 per seed.

### 3.4. Monitoring for Potential Harm

To monitor for potential harms and to improve our understanding of how appropriate and ethical network-based recruitment strategies are for use in South Africa given high levels of HIV-related stigma there, we conducted brief mixed methods follow-up interviews with participants approximately 6 weeks after baseline interviews. We asked participants (N = 55; TRIP arm = 12; TRIPLE arm = 43) about any negative, stigmatizing, or positive experiences they had during and/or due to study participation. All participants reported that they had *no* experiences of negative reactions from anyone they tried to recruit, and that their participation did not cause anyone to judge them or treat them badly, or to think they were HIV-positive. Most participants reported feeling comfortable recruiting others to the study (65% very comfortable; 15% somewhat comfortable). No significant differences were found in reported comfort by sex, study arm, or HIV status. All participants who completed follow-up interviews reported that their participation had helped them in some way. When asked how, 20% said that they enjoyed helping others get tested. In their responses to open-ended questions, some TRIPLE participants suggested that TRIPLE might help them deal with or avoid stigma by providing a way to urge partners to get tested without revealing their own HIV status.

## 4. Discussion

Even with all-female seeds, the study’s TRIPLE arm (which used expanded social-network-based recruiting) recruited and tested a high rate of male network members (53% men) for HIV testing. The TRIPLE arm tested males for HIV at a significantly higher rate than did the TRIP arm (25% men), which used traditional risk network recruiting. Furthermore, the TRIPLE arm recruited more male network members for HIV testing per seed than did the TRIP arm. These are extremely important findings since many clinics in South Africa test men at very low rates (e.g., 28% in local clinics) and since national models estimate that 32% of HIV infections among men in South Africa are undiagnosed, compared to an estimated 19% of infections among women [7].

These findings suggest that the TRIPLE approach of expanded social network tracing is a very promising intervention strategy for recruiting men to HIV testing who might not be reachable by standard risk network tracing. This may perhaps be due to increased male participant comfort discussing HIV testing with non-sex-partners. This potential explanation is supported by the fact that 40% of men in the TRIPLE networks were recruited by other men. This finding may suggest that male friends, relatives, and family members are a critical resource in getting reluctant men (e.g., those uncomfortable discussing testing with female sex partners; those concerned about stigma; those who believe that visiting a clinic to test is not consistent with masculine gender norms) to test. It may even be possible that male recruitment of other males could serve to directly address gender norm-related concerns about this issue if males become aware that their peers who they respect are getting tested for HIV and this reduces their perceptions of testing being outside of normative masculine behavior. However, a great deal of future research must be conducted that examines such potential processes before they can be considered anything other than speculation.

The TRIPLE approach also located previously undiagnosed HIV-positive men (and women) at higher rates per seed than did the TRIP approach. In other words, given a set of individuals who presented to clinics on their own for business-as-usual testing and were newly diagnosed with HIV, using an expanded social network-tracing approach (i.e., asking participants to recruit friends, family members, etc., who they thought might be at risk of being HIV-positive) resulted in the recruitment to HIV testing of more previously undiagnosed HIV-positive men than did traditional risk partner network- tracing. This provides further support for this approach as a promising new way of locating men.

These findings build upon the findings of the TRIP study conducted in Greece, Ukraine, and the United States, which modified traditional risk partner network tracing in a different way—by starting with seeds who were recently infected (in the last six months) with HIV—among concentrated HIV epidemics of people who inject drugs and of men who have sex with men. The TRIP study found that this method of HIV case finding was efficient and safe [15,16,17,18], and, along with other studies, comprises the scientific evidence that supports the inclusion of network-based approaches in the World Health Organization’s Consolidated Guidelines on HIV Testing Services [26]. Moreover, the TRIP study found that, between baseline and follow-up, participants’ perceptions and experiences of social support related to HIV and HIV-related services increased [27]. This finding suggested the possibility that the process of network-based recruitment and the peer discussions about HIV testing that it requires and engenders may actually improve social support among networks. While additional research is needed to test this potential mechanism directly, if it is true, then it stands to reason that the TRIPLE model, which expands HIV testing recruitment (and the peer conversations about HIV testing that facilitate such recruitment) to friends and family members, could have a potentially wider social reach for potential improvement of HIV-related social support and social support around testing. Again, future research should test this possible effect directly, as well as any potential effect that such improved social support may have on treatment cascade outcomes.

## 5. Limitations

The present pilot study was limited by its small sample size and, in particular, the small number of seed participants recruited to the study. The willingness and success of these seed participants in terms of recruiting their network members to HIV testing was promising and suggests that these interventions should be tested among a larger sample. The present study was also limited by its low external validity. We only recruited seeds from two Department of Health clinics in the study region, which has 55 Department of Health clinics. Our findings cannot be generalized to other populations, or even to other clinic sites in the study region. It is possible that patients newly diagnosed at some clinics would be less likely to be willing to participate in a network intervention soon after receiving an HIV diagnosis, for example. We are currently developing a future study to test network-based recruitment to HIV testing among a larger number of seeds recruited from a larger number of clinics. Another possible limitation of the present study is that it is possible that some seeds may not have truly been diagnosed for the first time but may instead have only told the clinic staff that they had never been diagnosed before. Patients may sometimes falsely present as new patients when they have had a lapse in their treatment and do not want to be scolded by healthcare providers for non-adherence. Department of Health clinics in South Africa do have electronic systems to track HIV testing and treatment, but these systems are not available in all clinics, and are also centrally managed such that it can take time for clinics to receive data on testing and treatment conducted by other clinics. It is, therefore, possible that some seeds were “re-diagnosed” before joining the study. However, even if this is the case, it should not affect our study arm comparisons since, in theory, randomization of seeds to study arm should result in equivalent seed characteristics between arms.

## 6. Conclusions

The present findings suggest that the TRIPLE approach is extremely promising for both recruiting men in KwaZulu-Natal to HIV testing and for locating undiagnosed HIV-positive men in this context and referring them to treatment. Given the known and widely discussed [7,28] challenges of testing and treating men for HIV in this context, the present findings suggest that the TRIPLE expanded social network-tracing approach should be tested formally using larger samples and multiple sites (e.g., via a randomized controlled trial or a site-randomized trial). If found to be efficacious among larger samples and across a range of settings in South Africa, and scaled up, this approach has the potential to reduce HIV transmission and increase progress towards 95–95–95 goals by reducing the number of undiagnosed HIV-positive men in South Africa and by improving linkage to care among men who have previously been reluctant to engage with HIV services.

## Figures and Tables

**Table 1:** Descriptive statistics of sample sociodemographic and socioeconomic characteristics.

	Full Sample of Seeds and Network Members (N = 85) Mean (SD) or Frequency (%)	TRIP Network Members (N = 12) Mean (SD) or Frequency (%)	TRIPLE Network Members (N = 62) Mean (SD) or Frequency (%)
Age	30.87 (11.03)	27.79 (8.43)	31.56 (11.76)
Employed	22 (25.88%)	3 (25.0%)	15 (24.19%)
Completed High School	35 (41.18%)	7 (58.33%)	26 (41.94%)
Primary Language is isiZulu	72 (84.71%)	11 (91.67%)	53 (85.48%)
Already HIV-positive-aware at Baseline *	2* (2.35%)	2* (16.67%)	0* (0.00%)

* Does not include seeds, all of whom were newly diagnosed just before joining the study.

**Table 2:** Comparison of TRIP vs. TRIPLE network-based recruitment to HIV testing intervention strategies in KwaZulu-Natal, South Africa.

Intervention Type	N Network Members (N Seeds)	Average Network Size	Newly Diagnosed Positives (NDP)	Additional NDP Located per Seed	Men Recruited
TRIPLE	62 (6 seeds)	10	9 (14.5%)	1.5	53.2%
TRIP	12 (5 seeds)	3	6 (50.0%)	1.2	25.0%
TRIP vs. TRIPLE χ^2^			χ^2^ = 7.8; *p* = 0.012		χ^2^ = 3.2; *p* = 0.073
“Business as usual” in nearby clinics			13% average		27.8% average

## Data Availability

Given the small number of participants, the relationships of participants to each other (i.e., participants’ knowledge of other people’s participation in the study due to the use of peer recruitment), and the sensitive nature of HIV test results, data are not available for sharing at the suggestion of the research ethics committee, in order to protect the privacy of participants.
